# The Candidate Schizophrenia Risk Gene *Tmem108* Regulates Glucose Metabolism Homeostasis

**DOI:** 10.3389/fendo.2021.770145

**Published:** 2021-10-08

**Authors:** Jianbo Yu, Xufeng Liao, Yanzi Zhong, Yongqiang Wu, Xinsheng Lai, Huifeng Jiao, Min Yan, Yu Zhang, Chaolin Ma, Shunqi Wang

**Affiliations:** ^1^ Laboratory of Synaptic Development and Plasticity, Institute of Life Science & School of Life Sciences, Nanchang University, Nanchang, China; ^2^ Department of Biology, Senior Middle School of Yongfeng, Ji’an, China; ^3^ School of Basic Medical Sciences, Nanchang University, Nanchang, China

**Keywords:** *Tmem108*, schizophrenia, glucose tolerance, insulin resistance, metabolism, metformin

## Abstract

**Background:**

Schizophrenia (SCZ) is a severe psychiatric disease affected by genetic factors and environmental contributors, and premorbid abnormality of glucose metabolism is one of the SCZ characteristics supposed to contribute to the disease’s pathological process. Transmembrane protein 108 (*Tmem108*) is a susceptible gene associated with multiple psychiatric diseases, including SCZ. Moreover, *Tmem108* mutant mice exhibit SCZ-like behaviors in the measurement of sensorimotor gating. However, it is unknown whether *Tmem108* regulates glucose metabolism homeostasis while it involves SCZ pathophysiological process.

**Results:**

In this research, we found that *Tmem108* mutant mice exhibited glucose intolerance, insulin resistance, and disturbed metabolic homeostasis. Food and oxygen consumption decreased, and urine production increased, accompanied by weak fatigue resistance in the mutant mice. Simultaneously, the glucose metabolic pathway was enhanced, and lipid metabolism decreased in the mutant mice, consistent with the elevated respiratory exchange ratio (RER). Furthermore, metformin attenuated plasma glucose levels and improved sensorimotor gating in *Tmem108* mutant mice.

**Conclusions:**

Hyperglycemia occurs more often in SCZ patients than in control, implying that these two diseases share common biological mechanisms, here we demonstrate that the *Tmem108* mutant may represent such a comorbid mechanism.

## Introduction

Schizophrenia (SCZ) is a severe, chronic, and heritable mental disorder that affects about 1% population. SCZ is characterized by negative symptoms (reduced emotional expression, increased cognitive deficits, and social isolation, et al.) and positive symptoms (excitatory status, delusions, and hallucinations et al.) (1–3). While the causes of SCZ are widely investigated, evidence demonstrates the etiology is multifactorial and prompts interaction between genetic factors and environmental contributors (1, 4). SCZ is typically present in the young population, and a study on twin and adoption families demonstrates that SCZ prefers to aggregate in families, suggesting the pivotal effect of genetic factors rather than environmental contributors (5–7).

The connection between SCZ and glucose dysregulation was illustrated to share a common field in pre-antipsychotic and post-antipsychotic years (8, 9). The premorbid abnormality of glucose metabolism is one of the SCZ characteristics attributed to the central insulin signaling pathway (8, 9). The connection between SCZ and hyperglycemia was reported as early as 1919, and then the correlations were described more and more. In 1926, glucose intolerance was declared to be more common in SCZ patients than in common individuals. The associations of SCZ with impaired insulin sensitivity were recorded before the antipsychotic introduction.

TMEM108 is a single transmembrane glycoprotein. It is linked with several psychiatric disorders in genome-wide association studies (GWAS), such as bipolar disorder (10–12), alcoholism (13), major depression (14), and SCZ (10, 11, 15–17). A single nucleotide polymorphism (SNP) (rs7624858) site, located in the intron of *Tmem108*, is linked to SCZ in GWAS (15).

*Tmem108* is expressed as early as E8.5 in the central nervous system (CNS) (18, 19) and is a multi-function molecular in a recent study (12, 20–22). TMEM108, also known as Retrolinkin (23–25), promotes endosomal vesicle retrograde transport in sensory neuronal axons by directly interacting with the dynastic/dynein cargo adaptor BPAG1n4 (25). TMEM108 in cultured hippocampal neurons cooperates with endophilin A1 through the proline-rich domain and participates in BDNF-induced dendrite outgrowth by mediating the BDNF-TrkB-ERK signaling pathway (24). Further research found that TMEM108 also recruits CYFIP1/2 units of the WAVE1 complex to facilitate the endocytosis of BDNF-TrkB in the hippocampal neuron (23).

Recent research found that TMEM108 maintains glutamatergic transmission and is required for spine development (16). The deletion of *Tmem108* weakens adult neurogenesis in the hippocampal dentate gyrus (22). *Tmem108* knockout impairs mouse immobility in a force swimming test and tail suspending test, and impairs space memory and contextual memory (16, 22). Furthermore, the mutant of TMEM108 induces deficits of pre-pulse inhibition (PPI) (16). PPI is a classic and plausible measurement in SCZ research (26). It is unknown whether TMEM108 simultaneously involves both SCZ pathological processing and plasma glucose metabolism homeostasis.

In this study, the role of *Tmem108* in plasma glucose level and metabolism homeostasis was invested firstly. *Tmem108* was expressed in the organs regulating plasma glucose metabolism, and *Tmem108* mutant mice exhibited glucose tolerance, insulin resistance, and disturbed-metabolic homeostasis. Food and oxygen consumption decreased, and urine production increased, accompanied by weak fatigue resistance in the mutant mice. Simultaneously, the glucose metabolic pathway was enhanced, and lipid metabolism decreased in the mutant mice, consistent with the elevated RER. Moreover, metformin attenuated plasma glucose levels and improved sensorimotor gating in *Tmem108* mutant mice. In a word, hyperglycemia occurs more often in the SCZ patients than controls, implying that these two diseases may share common biological mechanisms, here we demonstrate that the *Tmem108* mutant may represent such a comorbid mechanism.

## Materials and Methods

### Materials

*Tmem108* mutant (*Tmem108*-*LacZ*) mice (MMRRC: 032633-UCD) were described previously (16, 22), and briefly, β-galactosidase protein cassette, including both stop code and a polyadenylation termination signal, was replaced the first coding exon of *Tmem108* (exon 3) by homologous recombination. Mice were housed in a 12 h light/dark cycle room with ad libitum access to water and rodent chow diet. The animal protocols in this study were approved by Nanchang University Medical Sciences Committee for research on vertebrate animals, following EN Directive 2010/63/EU on the protection of animals used for scientific purposes. After terminal experiments, mice were euthanized by carbon dioxide inhalation.

### RNA Extraction and Real-Time PCR

According to the manufacturer’s instructions, the total RNA from mice tissues was extracted by TRIzol Reagent (Invitrogen, Thermo Fisher Scientific, USA). Real-time PCR was performed as described previously (27). The relative expression values were calculated relative to β-Actin by using the 2^-ΔΔCT^ methods, and the control group was set to a value of 1. In the end, the mutant group was normalized to the control. Primer specificity was tested by melting curve analysis, and the PCR product was verified by agarose gel electrophoresis. The real-time PCR primer sets are listed in **Table 1**.

**Table 1:** Primers sequences used in the real-time PCR assays.

Gene name	Forward primer sequence (5′–> 3′)	Reverse primer sequence (5′–> 3′)
*Acaca*	GTTCTGTTGGACAACGCCTTCAC	GGAGTCACAGAAGCAGCCCATT
*Gapdh*	CATCACTGCCACCCAGAAGACTG	ATGCCAGTGAGCTTCCCGTTCAG
*Gcg*	CCTTCAAGACACAGAGGAGAACC	CTGTAGTCGCTGGTGAATGTGC
*Hk*	GAAAGGAGACCAACAGCAGAGC	TTCGTTCCTCCGAGATCCAAGG
*Hmgcs*	GGAAATGCCAGACCTACAGGTG	TACTCGGAGAGCATGTCAGGCT
*Hmgcr*	GCTCGTCTACAGAAACTCCACG	GCTTCAGCAGTGCTTTCTCCGT
*Ide*	CAAACCTCTCCTTCCAAGTCAGC	TGTTCTCCGAGGTGCTCTGCAT
*Ins*	CGTGGCTTCTTCTACACACCCA	TGCAGCACTGATCCACAATGCC
*Igf*	GTGGATGCTCTTCAGTTCGTGTG	TCCAGTCTCCTCAGATCACAGC
*Pdges*	GTGGTTTCAGCAGGGTGTCACT	GTCTTGAGTCCAGATTTGCAGCC
*Pck*	GGCGATGACATTGCCTGGATGA	TGTCTTCACTGAGGTGCCAGGA
Tmem108	CCTGAGCTACTGGAACAATGCC	CAGTGTCTCGATAGTCGCCATTG

Acaca, Acetyl-CoA carboxylase alpha; Gapdh, Glyceraldehyde-3-phosphate dehydrogenase; Gcg, Glucagon; Hk, Hexokinase; Hmgcr, 3-Hydroxy-3-methylglutaryl-CoA reductase; Hmgcs, 3-Hydroxy-3-methylglutaryl-CoA synthase; Ide, Insulin-degrading enzyme; Ins, Insulin; Igf, Insulin-like growth factor; Pdges, Prostaglandin E; Pck, Phosphoenolpyruvate carboxykinase.

### Glucose Tolerance Test (GTT) and Insulin Resistance Test (IRT)

For GTT, after fasting the mice overnight for about 16 h, intraperitoneally (i.p.) glucose (2.5 mg glucose/g body weight) injection was performed on the mice. Then, plasma glucose concentrations were measured by a glucometer (Roche Accu-Chek Performa, USA) on each mouse before 20, 40, and 120 minutes after the i.p. glucose injection. Tail sipping was used to get blood. Before snips, the tail end was dipped into 0.25% bupivacaine for local anesthesia to reduce pain.

IRT was performed after a 6-h fast, and plasma glucose was measured before 20, 40, and 120 minutes after an i.p. insulin (0.75 mU/g body weight) injection.

### Metabolic Expenditure Measurements

Food intake, water consumption, feces outtake, and urine production were assessed by a metabolic cage (Tecniplast Metabolics Biological Monitoring, Italy). Briefly, 2-month-old male mice were individually housed in the metabolic cage 12 h light/dark cycle with ad libitum access to rodent chow diet and water. The data were collected by 12-h cycles for one month after a one-week adaption, and the data were normalized to the body weight.

Whole-body oxygen consumption (VO_2_), carbon dioxide expiration (VCO_2_), RER, and ambulatory activity were assessed by a Comprehensive Lab Animal Monitoring System (CLAMS, Columbus Instruments, USA). Briefly, 2-month-old male mice were acclimated to CLAMS cages individually with food and water ad libitum for 24 h. The data on oxygen consumption (VO_2_) and carbon dioxide production (VCO_2_) were recorded every 10 min for 3 d at 23°C following adaption. Infrared beams measured the ambulatory activity in a single horizontal plane (X-axis, Y-axis) and a vertical direction (Z-axis) (OPT-M3, Columbus Instruments). The RER was calculated as VCO_2_/VO_2_ automatically by the software.

### Open Field Test

Male mice were handled for habituation for 3 d before behavioral tests. The open field was a square chamber (40 × 40 × 30 cm). Mice were placed in the chamber individually and monitored for 10 minutes with an overhead video tracing system to record the animal’s locomotor activity.

### Rotarod Wheel Walking

Mice balance and motor coordination were measured with a rotarod apparatus (Unibiolab, Beijing, China). The first day was used for acclimation training, which consisted of two trials with a 15-min resting interval between two trials. The first trial was performed at a 13-rpm speed for 15 min, and the speeds in the second trial ramped from 13 rpm to 25 rpm in 15 min. After acclimation training, mice were used for the test trial, including speeds ramping from 13 rpm to 25 rpm in 15 min and maintaining 25 rpm for 30 min. Mice were resumed on the rotarod if they stopped running or fell off four times within 60 s of each fall. The latency falling off the rotarod of mice was recorded individually.

### Muscle Fatigue Task Test

A muscle fatigue task test was performed on a six-lane treadmill (Columbus Instruments, USA). The speed was accelerated with a specified ramp from the starting speed of 0 m/min to the maximum speed of 20 m/min. Mice ran until exhaustion. Definition of the exhaustion was the mice’s inability to restart running by continuous contact (no less than 5 sec) even with the electrical shock at the end of the running lane. The running time of each mouse was recorded. Moreover, the distance was calculated based on the treadmill’s speed and the mouse running time.

### Pre-Pulse Inhibition (PPI)

Pre-pulse inhibition (PPI) was performed in sound-attenuated chambers (Med Associates Inc., St. Albans, VT, USA) as previously described (16) with minor revision. In brief, a 65-dB background whiter noise was maintained throughout the test sessions. Mice were acclimated to the testing chamber for 5 min as the first test session. Then, mice were individually placed in a tube fixed on a plastic frame to monitor movement. After 12 initial startle pulses (20 ms, 120 dB) were presented, 12 prepulse/startle trials (prepulse, 20-ms white noise at 70, 80, or 90 dB at 100-ms intervals; 80-ms interval; startle pulse, 20 ms,120 dB) were performed immediately. The initial startle pulses provided a baseline of startle reactivity for the following test session. The different trial types (12 times for each trial type) were presented pseudo-randomly with a 15-s inter-trial interval (10-20 s), and two consecutive trials were always different. Mice movements were analyzed for 100 ms from startle stimulus onset. Each complete test session lasted about 25 min. For each pre-pulse intensity, PPI (%) was calculated as: [1 - (startle amplitude on “pre-pulse followed by pulse” trial/startle amplitude on “pulse-alone” trial)] × 100.

### Experimental Design and Statistical Analysis

The study was not pre-registered. For the assignment of each experimental group, no unique randomization method was applied. Sample sizes were determined by reference and experience. The experiments reported in this study did not require institutional approval. Exclusion criteria were not employed in this research. All Data statistics were conducted by GraphPad Prism 6.0 (GraphPad Software Inc, USA), and the values were mean ± SEM. Different people performed the analysis and experimental group assignments than the experimenter. As indicated in the figure legends, the data were analyzed by two-way ANOVA or unpaired t-test where appropriate. Significance between two groups were indicated as * p*<*0.05, ** p*<*0.01 or *** p*<*0.001, and p > 0.05 was considered no significant.

## Results

### *Tmem108* Expression Profile in Several Indicated Tissues in Mice

*Tmem108* expression was verified by qPCR (**Supplementary Figure S1**) in several indicated tissues (brain, liver, muscle, and pancreas) in adult male wild-type mice, which are mainly related to glucose metabolism. The muscle and liver are the main mobilization organ of stored glycogen, which provide the most rapid and available glucose source for themselves and other organs. The pancreas maintains the plasma glucose balance by different exocrine hormones, such as insulin and glucagon, and diabetes is a disability of pancreas function. The brain constitutes no more than 5% of the body weight. However, except for acetone body utilization, the brain’s energy consumption accounts for 20%-30% of the total glucose utilization.

Moreover, the hypothalamus in the brain could secrete insulin (central insulin) to regulate glucose levels. *Tmem108* was widely expressed in mice’s indicated tissues, highly in the brain (including the hypothalamus, hippocampus, prefrontal cortex, olfactory cortex, and medulla) and liver, lowly in pancreas and muscle.

### The Altered Body Growth Curve in *Tmem108* Mutant Mice

*Tmem108* mutant mice reached adulthood were fertile and exhibited no apparent health issues, including average brain size and brain weight (**Supplementary Figure S2**). We performed the growth curve analysis to characterize the growth rates of *Tmem108* mutant mice (**Supplementary Figure S2**). *Tmem108* mutant mice were significantly smaller than the control (Wild-type) mice at P50 and P100. The growth curve difference was significant between *Tmem108* mutant mice and the control mice, indicating the slow growth rate in the *Tmem108* mutant mice during the postnatal period.

### Impaired of Glucose Homeosis in *Tmem108* Mutant Mice

**Figure 1:**
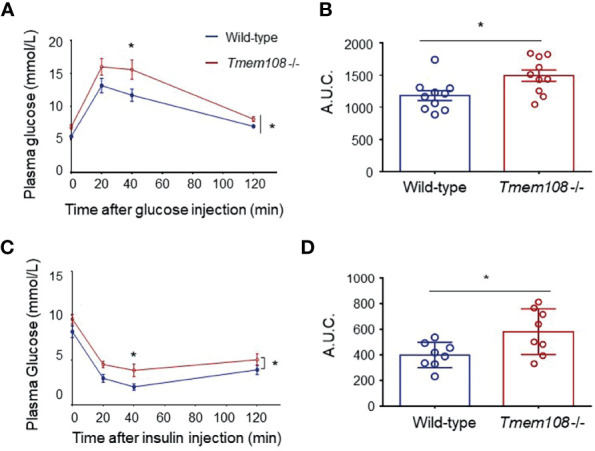
Impaired of glucose homeosis in *Tmem108* mutant mice. **(A)** Time courses of plasma glucose levels after overnight fasting in glucose tolerance test (GTT). Plasma glucose measured in *Tmem108* mutant (*Tmem108* -/-) mice was significantly higher than in control mice (wild-type). Glucose values in nmol/L mean ± SEM at designated time points after glucose bolus injection at time point 0 (n = 10 per group, two-way ANOVA). **(B)** AUC (area under the curve) for plasm glucose (0–120 min) was calculated during the GTT (unpaired *t*-test). **(C)** Time courses of plasma glucose levels after 4-hour fasting in insulin resistance test (IRT). Plasma glucose measured in *Tmem108* mutant mice was significantly higher than in control mice. Glucose values are means ± SEM at designated time points after insulin injection at time point 0 (n = 8 per group, two-way ANOVA). **(D)** AUC for plasm glucose (0–120 min) was calculated during the IRT (unpaired *t*-test). (*p < 0.05).

Carbohydrate metabolism was investigated in *Tmem108* mutant mice using glucose tolerance (GTT) and the insulin resistance test (IRT). A GTT is used to measure the efficiency of sugar metabolism. When the sugar is metabolized abnormally, plasma glucose levels will be too high or too low to maintain homeostasis, resulting in diabetes or hypoglycemia. Insulin is essential to process food’s sugars to energy, and an IRT is used to analyze the body’s response to the insulin hormone. Plasma glucose level is the same indicator in both GTT and IRT, and a similar analysis is a calculation of the area under the curve (AUC), which depends on integrating glucose values in the GTT or IRT. *Tmem108* mutant mice exhibited slower glucose clearance after bolus injection of glucose (**Figures 1A, B**) and slower clearance following insulin injection (**Figures 1C, D**), which means a lowered response to the insulin hormone in the mutant mice. This result indicated that *Tmem108* mutant mice exhibited impaired glucose homeosis, with metabolized glucose more slowly and higher insulin resistance than the control mice.

### A Shift of Metabolism Homeostasis in *Tmem108* Mutant Mice

**Figure 2:**
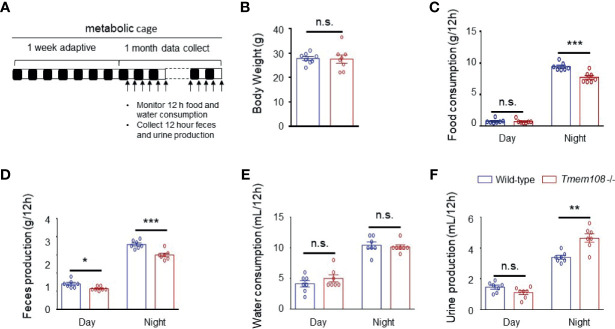
*Tmem108* deficiency changed food and water metabolic level. **(A)** Schematic diagram for the collection of excreta and monitoring of food/water consumption. Alternating white and black bars show the alternate of day and night. **(B)** Body weights of 3-month mice were not affected by *Tmem108* mutant (*Tmem108* -/-), wild-type mice as the control (n = 8 per group). **(C–F)**. The measurement was obtained from metabolic cage experiments. Food **(C)** and water consumption **(E)** and feces **(D)** and urine production **(F)** are shown as means ± SEM, which were normalized to body weights (n = 7 per group, unpaired *t*-test, *p < 0.05, **p < 0.01, ***p < 0.001). n.s., not significant.

We utilized simple metabolic cages for the tests over one month to detect the mice’s consumption and production (**Figure 2A**). Compared to the control mice, food intake and feces excretion were essentially lower in *Tmem108* mutant mice (**Figures 2C, D**). Furthermore, water consumption was identical in the two mice (**Figure 2E**), and urine production in *Tmem108* mutant mice was slightly higher in the night than in the control mice (**Figure 2F**). However, there were no differences in the weight between *Tmem108* mutant mice and the control mice (**Figure 2B**). The results suggested that *Tmem108* deficiency in the mutant mice might shift the metabolism homeostasis unless the mutant mice have severe issues in the metabolic architecture.

**Figure 3:**
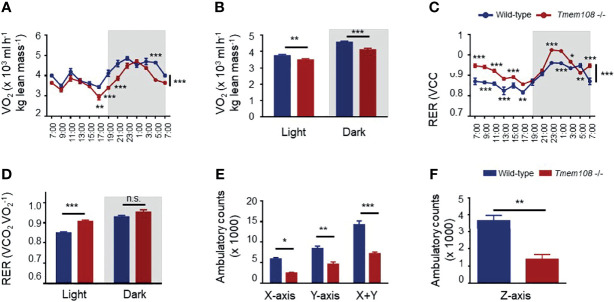
Energy metabolism was remodeled in *Tmem108* deficiency mice. **(A)** Determination of VO_2_ in *Tmem108* mutant (*Tmem108* -/-) mice over a 24-h period, normalized to body weight, wild-type mice as the control. **(B)** Consumption of VO_2_ in *Tmem108* mutant mice in 12-light and dark cycles was analyzed. **(C)** Determination of respiratory exchange ratio (RER) in *Tmem108* mutant mice over a 24-h period. **(D)** RER of *Tmem108* mutant mice in 12-h light and dark cycles were analyzed. **(E)** Ambulatory counts of mice in X-axis or Y-axis or both. **(F)** Ambulatory counts of mice in Z-axis. (Values are mean ± SEM, n = 8 per group, male mice; two-way ANOVA were performed between two curves in panel A and C; other statistics were unpaired *t*-test; n.s., not significant, *p < 0.05, **p < 0.01, ***p < 0.001).

A comprehensive laboratory animal monitoring system was also used to record the mice’s whole-body oxygen consumption and carbon dioxide production and measure the ambulatory movements in similar home-cages. Oxygen consumption (VO_2_) was substantially lower in *Tmem108* mutant mice than wild-type, both in the light and dark phases (as expected for nocturnal animals, **Figures 3A, B**). The mice were cultured in the metabolism cages for 3 d, and the data of the last 24 h were used for analysis. The RER, also known as a respiratory quotient (RQ), is the volume ratio of carbon dioxide released to oxygen absorbed during respiration. The results showed that RER in *Tmem108* mutant mice was higher than in the control mice in light (**Figures 3C, D**), indicating the mutant mice preferred consuming more carbohydrates or fewer lipid compounds than the control mice. Furthermore, *Tmem108* mutant mice might have a low metabolism level because of less oxygen consumption in the mutant mice.

The locomotor activity of mice in the metabolism cages was represented by ambulatory counts (**Figures 3E, F**). Ambulatory counts of *Tmem108* mutant mice were lower than the control mice in three axes, which meant horizontal locomotion (Ambulatory counts in X-axis and Y-axis) and vertical exploration (Rearing behavior, ambulatory counts in Z-axis) were reduced in *Tmem108* mutant mice (**Figure 3F**). The reduction in ambulatory counts suggested a significant inhibitory/abnormal effect in *Tmem108* mutant mice.

### Expression Profile of the Glycolytic and Lipogenic Genes in *Tmem108* Mutant Mice

**Figure 4:**
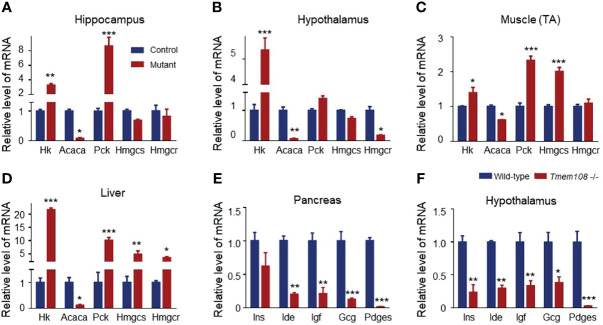
Expression profile of the metabolic and hormone genes in *Tmem108* deficiency mice. **(A–D)** The glycolytic and lipogenic gene relative expression in several tissues was qualified by Real-time PCR, including the hippocampus **(A)**, hypothalamus **(B)**, tibialis anterior (TA) muscle **(C)**, and Liver **(D)**, with Gapdh as the internal control. *Hk* increased, and *Acaca* decreased in the detected tissues of the mutant mice (*Tmem108* -/-), wild-type mice as the control. **(E, F)**. The hormone gene is related to metabolism in the pancreas **(E)** and hypothalamus **(F)**. Most of the detected hormone gene mRNA levels decreased in the mutant mice. (n = 5 per group, male mice; total RNA from each mouse tissue was pooled to generate cDNA. Data are represented as means ± SEM; two-way ANOVA Sidak’s multiple comparisons test, *p < 0.05, **p < 0.01, ***p < 0.001).

RER in *Tmem108* mutant mice was higher than in control mice, indicating a high glucose oxidation ratio to lipid metabolism. Therefore, the relative expression of the rate-limiting enzyme of glycolysis (*Hk*) and glycogenesis (*Pck*) in glucose metabolism were investigated (**Figures 4A–D**), an increase of both them indicating high carbohydrate metabolism in the mutant mice, consistent with the high RER of the mutant mice. Moreover, relative expression of the rate-limiting enzyme in lipid metabolism also was determined by qPCR, including *de novo* lipogenesis crucial gene (*Acaca*), cholesterol synthesis rate-limiting enzyme (*Hmgcr*), and acetone synthesis critical gene (*Hmgcs*). Strikingly in the mutant mice, Acaca expression decreased in all the detected tissues, indicating a low level of lipid metabolism.

Furthermore, several hormone-associated genes (*Gcg, Ide, Ins, Igf*, and *Pdges*) regulating plasma glucose levels and lipid metabolism were also verified in the pancreas and hypothalamus (**Figures 4E, F**). mRNA of the detected hormone gene-regulating glucose and lipid metabolism decreased in the mutant mice.

### Impaired Fatigue Resistance of *Tmem10*8 Mutant Mice Without Deficiency of Motor Coordination Ability

**Figure 5:**
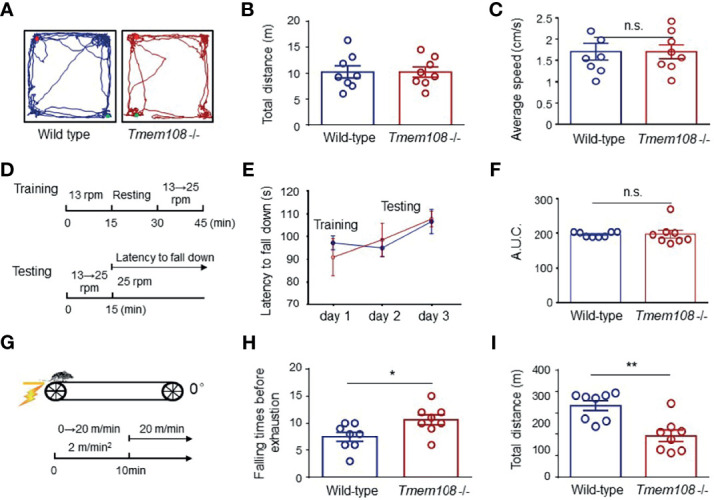
Impaired fatigue resistance of *Tmem108* mutant mice without deficiency of motor coordination ability. **(A)** Representative traces of the mice in the open field test. The mouse was placed in a chamber, and the movement was monitored for 10 min (n = 8 per group, male mice). **(B)** Similar total distance traveled between *Tmem108* mutant (*Tmem108* -/-) mice and the control mice (Wild-type). **(C)** The average movement speed in *Tmem108* mutant mice was not different from the control mice. **(D)** Diagram of the rotarod test. The mouse was put on a rotarod wheel. Day 1 was for acclimation training, and there were two trials each day for training or testing. In the second trial, mice were resumed on the rotarod when they stopped running or fell off 4 times within 60 s of each fall, and the latency to fall was recorded individually (n = 8 per group, male mice). **(E)** The latency to fall was similar in training or testing days between two types of mice. **(F)** AUC for the latency to fall (day 1-3) was calculated during the rotarod test. **(G)** Diagram of muscle fatigue task test. The speed was accelerated with a 2 m/min*min ramp 0 m/min to 20 m/min. The mice’s exhaustion was unable to restart by continuous contact (≥ 5 sec) even with the electrical shock (n = 8 per group, male mice). **(H)** The falling times of mice before exhaustion was recorded individually. **(I)** The total distance for each mouse was calculated by the product of speed and the corresponding time. (Data are represented as means ± SEM; unpaired *t*-test, *p < 0.05, **p < 0.01). n.s., not significant.

Before the mice were used for the muscle fatigue test, the mice’s locomotor activity and balance ability were assessed by open field test and rotarod test separately. Firstly, *Tmem108* mutant mice have no difference from the control mice in the total travel distance and the average speed evaluated by the open field test (**Figures 5A–C**). And then, in the rotarod wheel test (**Figures 5D–F**), *Tmem108* mutant mice have similar performance with the control mice in the balance learning and keeping. Therefore, damnify of motor coordination ability was not evidenced in *Tmem108* mutant mice. Intriguingly, *Tmem108* mutant mice manifested weak fatigue resistance in the muscle fatigue task test (**Figures 5G–I**) because the mutant mice had quickly exhausted when running on the treadmill.

### Metformin Slightly Enhanced Startle Responses of *Tmem108* Mutant Mice

Pre-pulse inhibition (PPI) is a psychophysiological measurement of sensorimotor gating. The startle reflex is suppressed when the startling stimulus is preceded by a subthreshold stimulus (28, 29). PPI is consistently standard in rodents and humans (28). PPI in *Tmem108* mutant mice decreased, as described before (16).

**Figure 6:**
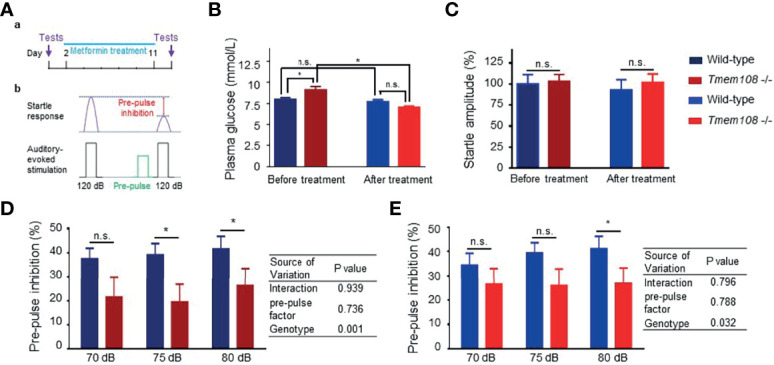
Metformin enhanced startle responses of *Tmem108* mutant mice. **(A)** Diagram of pre-pulse inhibition (PPI) test. The response to auditory-evoked startle stimulus (120 dB) was conducted. PPI test was introduced again after ten-day treatment of metformin. **(B)** Baseline of plasma glucose level was ameliorated after metformin treatment, wild-type as the control. **(C)** Baseline startles responses were similar between the two genotypes of mice. **(D, E)**. PPI was measured in both types of mice before or after metformin treatment. Compared with the control mice, reduced PPI in *Tmem108* mutant mice was found at pre-pulse (75 dB and 80 dB) before metformin treatment **(D)**, and PPI decrease in *Tmem108* mutant mice was found only at pre-pulse 80 dB after metformin treatment **(E)**. (n = 8 per group, male mice; data are represented as means ± SEM; unpaired *t*-test, n.s., not significant, *p < 0.05).

Metformin, the most commonly prescribed medication to treat diabetes (30, 31), was executed to treat the aberrancy of glucose metabolism in *Tmem108* mutant mice (**Figure 6A**). Before metformin treatment, plasma glucose levels in *Tmem108* mutant mice were higher than in the control mice. Intriguingly, metformin-induced a decline of plasma glucose levels in *Tmem108* mutant mice significantly (**Figure 6B**). Whether treatment with metformin or not, the baseline response to auditory-evoked startle stimulus (120 dB) was no different between *Tmem108* mutant mice and the control mice (**Figure 6C**).

**Figure 7:**
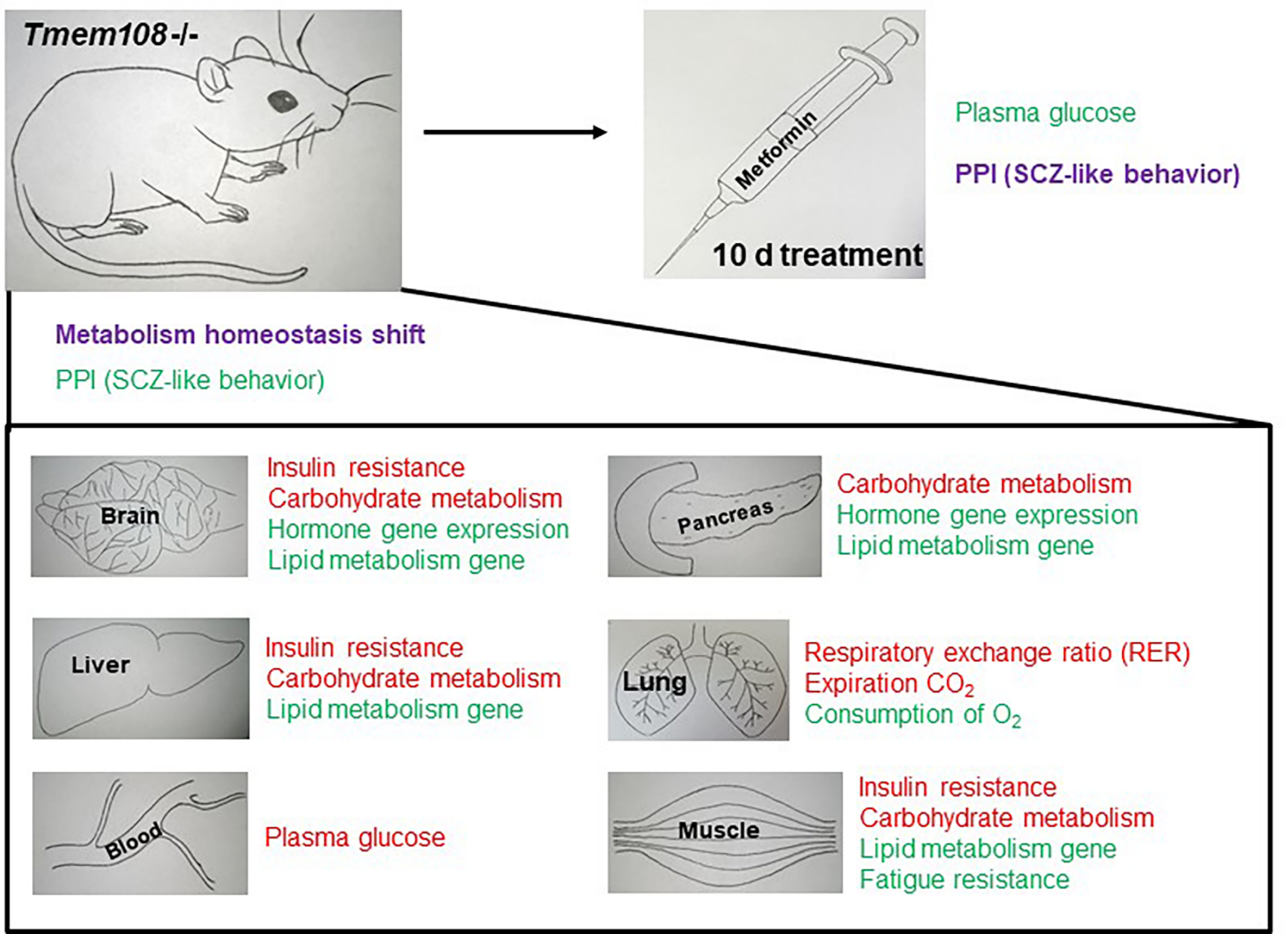
Summary of metabolism homeostasis shift and PPI change in *Tmem108* mutant mice. The phrase in red color means to increase, and the phrase in green color means decrease. Metabolism homeostasis has a complicated change, and PPI is slightly enhanced after metformin treatment for 10 d.

After metformin treatment for ten days, PPI in *Tmem108* mutant mice was lower than the control mice at pre-pulse 75 dB and 80 dB (**Figure 6D**). However, after ten-day metformin treatment, PPI decreased *Tmem108* mutant mice were found only at pre-pulse 80 dB (**Figure 6E**). Therefore, metformin slightly enhanced startle responses of *Tmem108* mutant mice and the enhancement was also found in the two-way ANOWA analysis, genotype variance variation from 0.001 (before metformin treatment, **Figure 6D**) to 0.032 (after metformin treatment, **Figure 6E**).

## Discussion

### *Tmem108* Is One of the Susceptible Genes in SCZ

There is a wide array of different mechanisms for illuminating the SCZ pathogenesis, including but not limited to neurodevelopmental deficits in the brain circuity, defects in one or more neurotransmitters, viral infection, and multifactorial inheritance (32). Rates of SCZ are higher in patients’ relatives than in common individuals. Studies on twins and adoption declare a tenfold increase in risk associated with the affected first-degree family. However, SCZ genetic transmission is not following Mendelian single-gene inheritance patterns, and it probably owes to multiple risk genes acting with environmental contributors. While their heritability has built up some genetic factors in SCZ, several genetic factors and environmental contributors may be sufficient to provoke the outbreak of SCZ (5–7). *Tmem108* is not only a susceptible gene of SCZ (10, 11, 15–17) but also a risk gene of other psychotic disorders, such as bipolar disorder (10–12) and major depressive disorder (14), which suggest *Tmem108* have a complicated role in the psychotic pathology.

For SCZ, genetic factors from the family are one of the leading causes, implying at least one risk gene in this family (33, 34). However, environmental factors also contribute to the onset of SCZ (35, 36), and the environmental factors take effect because of the risk gene existence, such as *Tmem108*. The environmental factors may take part in the diseases through many routes, directly or indirectly interacting with the risk genes (33, 34, 37), and whether direct or indirect interaction between *Tmem108* and SCZ is unclear.

### Plasm Glucose Level Dysregulation in *Tmem108* Mutant Mice and Antipsychotic Medication in Patients of SCZ

Comparing with the control mice, *Tmem108* mutant mice exhibited higher glucose intolerance in GTT and had higher plasma glucose levels before metformin treatment. In some reports, dysregulated cerebral glucose metabolism is regarded as a beginning matter of SCZ pathogenesis, and it causes the secondary alteration in peripheral glucose metabolism. The immunity may be associated with the inauguration in SCZ pathogenesis, causing abnormality in glucose metabolism. Common plausible elements may be accountable for the co-occurrence of glucose abnormality and immune disturbances in SCZ (38).

The blood glucose is affected in the *Tmem108* mutant mice. From the expression of glycolytic and lipogenic genes, muscle is one of the biggest organs in the body (39), and it is a major contributor to glucose homeostasis (40, 41). Though the brain constitutes no more than 5% of the body weight, the brain’s energy consumption accounts for 20%-30% of the total glucose utilization. So, brain contribution in blood glucose is not negligible. The pancreas and hypothalamus are upstream in glucose regulation (42–44), contributing more.

Abnormal glucose homeostasis emerges from the illness onset in SCZ, indicating that SCZ patients are at high risk of diabetes mellitus (DM) before medications (45). The co-occurrence of DM and SCZ was already documented in the 19th century. It is long before the occurrence of antipsychotic medications. Moreover, at that time, the propensity of diets could not induce metabolic derangements (45).

There are two main types of DM. Type I DM is caused by the damage of the pancreatic β cells. 90% of DM belongs to type II DM, accompanying insulin resistance and disruptive secretion (46). Rates of type II DM in SCZ are estimated to be 10% to 15%, much higher than that in ordinary individuals, and antipsychotic medication adds complexity to this correlation by adding DM’s risk (45, 47). Intriguingly, studies in SCZ patients’ relatives have disclosed elevated prevalence of DM similar to the result from a meta-analysis of the first episode psychosis (9, 45, 48, 49).

Treatments for SCZ mainly include typical antipsychotics emerging in the 1950s (also as first-generation antipsychotics) and atypical medication (known as second-generation antipsychotics, SGAs) 1980s. However, in recent years, the metabolic side-effects of SGAs have been substantially reported, such as impaired glucose regulation and manifest DM (50). The asymptomatic preclinical phase of DM is also distinguished by impaired glucose regulation and metabolism dysregulation. Some recent studies (51) have also advocated a potent high risk of DM in first episode SCZ populations before atypical antipsychotic administration, which may be associated with diabetogenic lifestyle factors and a genetic predisposition for DM (51). Atypical treatments replace typical treatments of SCZ because of extrapyramidal side effects and inadequate therapeutic potential (52). Although second-generation treatment of SCZ is secure and effective for movement disorders, high-risk metabolic side effects are related to them, which underly fasting hyperglycemia, impaired glucose tolerance, prediabetes, and diabetes (53). Antipsychotic medications are associated with severe metabolic abnormalities and fail to remedy premorbid cognitive deficits, though they are the cornerstone treatments for SCZ (9).

### Insulin Resistance in *Tmem108* Mutant Mice and Central Insulin Resistance in SCZ Patients

*Tmem108* mutant mice exhibited high insulin resistance in IRT. Central insulin resistance is the intersection of cognitive and metabolic diseases (54). Central insulin can mediate striatal dopamine levels (55), and striatal dopamine and central insulin can manage peripheral glucose metabolism (56–58). From a comprehensive analysis of 14 studies composing 1345 individuals, Toby Pillinger et al. found that participants with the first-episode SCZ exhibited high-level fasting plasma glucose and high insulin resistance (45). It is still difficult to establish the direct influence of atypical antipsychotic medication on glucose metabolism. However, short-term dosing research has suggested that antipsychotic medication involves weakened insulin sensitivity and impaired glucose tolerance (51). An insulin signaling pathway is impaired in first-episode psychosis patients and siblings (59–61), and the intriguing correlation between SCZ and DM is always a fruitful and active inquiry (9).

### *Tmem108* Expression and Insulin Regulation in the Brain

The highest active organ in whole-body metabolism is the brain (38). The brain consumes 20% of the whole oxygen supply and half of the whole glucose supply, although the brain-to-body mass ratio is no more than 5%. Due to the high energy consumption, cerebral glucose supply must be continuous, while energy storage substance is limited in the brain. Instead, ketone bodies and lactate can also be consumed as an energy supply in the brain. Ketone bodies originate from fatty metabolism, and lactate is the product of anaerobic glycolysis. Insulin and Insulin receptors are distributed through the CNS, not limited to be expressed in the hypothalamus (62).

*Tmem108* was widely expressed in the brain, including the hypothalamus, which was considered one area secreting insulin to regulate glucose levels. Therefore, we explored several hormone gene expressions adjusting glucose metabolism in the hypothalamus. The results showed that hormone genes associated with glucose and lipid metabolism were abated in the mutant mice.

The CNS insulin origin continues to be an argument between central and peripheral origins. In 1967, evidence showed that peripheral insulin infusion led to an insulin elevation in cerebrospinal fluid, suggesting peripheral insulin can pass through the blood-brain barrier (BBB). Insulin is transported to cross the BBB by the receptor-mediated system, such as N-methyl-D-aspartate receptors (NMDARs) and cannabinoid receptor type 1(CB1) (57, 63, 64). Evidence for the central origin of insulin is that some neurons can synthesize and secrete insulin due to the appearance of insulin mRNA and C-peptide in the brain and the release of insulin by *in vitro* primary neuronal cell cultures (9). Insulin expression decreased in *Tmem108* mutant mice.

Furthermore, central insulin involves about 50% of glucose metabolism in the brain. It is reasonable that insulin signaling dysfunction should be responsible for the cognitive deficits because the dysfunction occurs before antipsychotic administration. It is distinct that glucose uptake partly depends on central insulin in some brain regions (57). Insulin resistance is related to impaired glucose utilization (65). CNS insulin resistance may emerge with alteration in metabolic health. Hence, increasing insulin availability can plausibly improve cognitivism and metabolism (66).

### Decrease of Food and Oxygen Consumption in *Tmem108* Mutant Mice and Metabolism Regulation by Insulin

Food and oxygen consumption in *Tmem108* mutant mice were lower than the control mice, but urine production was higher, consistent with the elevated RER in *Tmem108* mutant mice. Insulin performance in the CNS is the primary regulator of cognitive processes and energy balance (9). Insulin resistance is related to change in homeostatic signals impacting glucose metabolism and food intake. Evidence has underlined a role for antipsychotic medication in regulating central insulin signaling pathways (9). Cerebral insulin performs well in the hypothalamus and modulates glucose homeostasis, food intake, and cognitive processes (67–69). SCZ is conceptualized as an illness of dopaminergic dysfunction, links to abnormal metabolism (particularly type II DM), and may share plausible overlaps with CNS insulin dysregulation because of premorbid cognitive deficits (9). The mechanisms for interpreting these effects are largely unknown (9). Central insulin signaling represents a fundamental relation between cognitive and metabolic diseases (9).

### Locomotor Activity and Fatigue Resistance in *Tmem10*8 Mutant Mice

*Tmem108* mutant mice manifested weak fatigue resistance in the muscle fatigue task test. Before the test, the locomotor activity and the balance ability were assessed by the open field test and rotarod test separately. The results suggested that *Tmem108* mutant mice have no difference from the control mice in the two types of tests. However, ambulatory counts representing locomotor activity were lower in *Tmem108* mutant mice than in the control mice in the metabolism cages. It looks weird that locomotor activity was not inconsistent in different behavior tests, resulting in the difference of accustomed time before recording the data. The mice were accustomed for a few minutes in the open field test, so movements involved novelty exploration. However, the mice were cultured in the metabolism cages for 3 days, including 2 days accustomed, and the collecting data reflected living status like the home-cages.

### Metformin Treatment in *Tmem108* Mutant Mice and SCZ

It is known that defective PPI is seen in multiple neuropsychiatric disorders, including SCZ, where reduced gating is believed to be a possible neurobiological mechanism undertaking some essential abnormal cognitive functions associated with this disorder (29). PPI is a classic and plausible measurement in SCZ research (26). In this research, although metformin induced a decline of plasma glucose levels in *Tmem108* mutant mice significantly, metformin addition slightly improved sensorimotor gating of *Tmem108* mutant mice.

Besides PPI in SCZ, cognitive deficits/impairment are also a hallmark of SCZ (70). Cognitive dysfunction risk factors may contribute to cognitive impairment in SCZ (70). SCZ with comorbid diabetes was more cognitively impaired than SCZ without diabetes or diabetes only, especially in working memory and attention (70–74). Diabetes-related factors including insulin resistance, hyperglycemia, and lipid metabolic disorders may affect cognitive function (54, 75, 76). The levels of insulin resistance are elevated and correlated with the severity of cognitive impairment in first-episode SCZ patients (77–79).

Metformin is an effective treatment of prediabetes, and herein, it can attenuate the metabolic abnormality induced by antipsychotic medication without abridging the therapeutic effects (52, 53, 80). Metformin can reverse MK-801 induced schizophrenia-like symptoms, such as deficits, anxiety-like symptoms, hyperactivity, and spatial memory impairment (81).In SCZ treatment, metformin addition can improve physiological parameters and behaviors. SCZ frequently accompanies depression, with a rate of 20%-60% depending on the pathology phase (52, 82–84). Metformin can enhance antipsychotic, antidepressant, and anxiolytic activities (52, 62, 85).

## Conclusions

*Tmem108* mutant mice exhibited high glucose intolerance, insulin resistance, and disturbed metabolism homeostasis with the elevated respiratory exchange ratio (RER). Furthermore, metformin ameliorated plasma glucose level and improved sensorimotor gating of the mutant mice. In brief, *Tmem108* mediated metabolic homeostasis and regulated plasma glucose metabolism. Hyperglycemia occurs more often in SCZ patients than controls, implying that these two diseases share common biological mechanisms, here we demonstrate that *Tmem108* mutant may represent such a comorbid mechanism.

## Data Availability Statement

The raw data supporting the conclusions of this article will be made available by the authors, without undue reservation.

## Ethics Statement

The animal study was reviewed and approved by Nanchang University Medical Sciences Committee.

## Author Contributions

SW initiated and designed the study. JY and XFL performed a glucose tolerance test, insulin resistance test, behavior test, and qPCR. JY and YZZ performed the metabolism test. YW and YZ cultured mice and assisted in the animal test. XSL and CM analyzed data. HJ and MY advised on the project. SW wrote the manuscript with input from all coauthors. All authors contributed to the article and approved the submitted version.

## Funding

This work was supported partly by grants from the National Natural Science Foundation of China (31760276, 31960171, 81860242, 31460260) and the Jiangxi Natural Science Foundation (20171BAB204019, 20192ACB20022).

## Conflict of Interest

The authors declare that the research was conducted in the absence of any commercial or financial relationships that could be construed as a potential conflict of interest.

## Publisher’s Note

All claims expressed in this article are solely those of the authors and do not necessarily represent those of their affiliated organizations, or those of the publisher, the editors and the reviewers. Any product that may be evaluated in this article, or claim that may be made by its manufacturer, is not guaranteed or endorsed by the publisher.

## Supplementary Material

Supplementary Figure 1
*Tmem108* expression profile in several indicated tissues in mice. A. Cartoon indicating TMEM108 domains in amino acid sequence. B. Relative expression *Tmem108* in indicated tissues in 3-moth-old wild-type mice by Real-time PCR, Gapdh expression as internal control, *Tmem108* relative expression in the pancreas was defined as one, wild-type mice, n=5.

Supplementary Figure 2 Altered body growth curve of *Tmem108* mutant mice. A. Representative brain figures of the adult mice (2 months). B. No alteration of brain weight in *Tmem108* mutant (*Tmem108* -/-) mice, wild-type mice as the control (Values are mean ± SEM, n = 12 2-month-old mice per group, unpaired *t*-test, n.s., not significant). C. Growth of *Tmem108* mutant mice was slower than the control mice. (mice per group, n = 12, Sidak’s multiple comparisons test was used to compare the two groups accompanying with two-way ANOVA, *p < 0.05, **p < 0.01).

## Abbreviations

*Acac*, Acetyl-CoA carboxylase; BBB, Blood-brain barrier; DM, Diabetes mellitus; *Gapdh*, Glyceraldehyde-3-phosphate dehydrogenase; *Gcg*, Glucagon; GTT, Glucose tolerance; GWAS, Genome-wide association studies; *Hmgcr*, 3-Hydroxy-3-methylglutaryl-CoA reducase; *Hmgcs*, 3-Hydroxy-3-methylglutaryl-CoA synthase; *Hk*, Hexokinase; *Ide*, Insulin-degrading enzyme; *Ins*, Insulin; *Igf*, Insulin-like growth factor; IRT, Insulin resistance test; *Pdges*, Prostaglandin E; *Pck*, Phosphoenolpyruvate carboxykinase; PPI, Prepulse inhibition; RER, Respiratory exchange ratio; RQ, Respiratory quotient; SCZ, Schizophrenia; SNP, Single nucleotide polymorphism; *Tmem108*, Transmembrane protein 108.
