# Supplementary figures and images for: The Candidate Schizophrenia Risk Gene Tmem108 Regulates Glucose Metabolism Homeostasis
Source: Front Endocrinol (Lausanne). 2021 Oct 8;12:770145. doi: 10.3389/fendo.2021.770145 (PMC8531597; doi:10.3389/fendo.2021.770145)

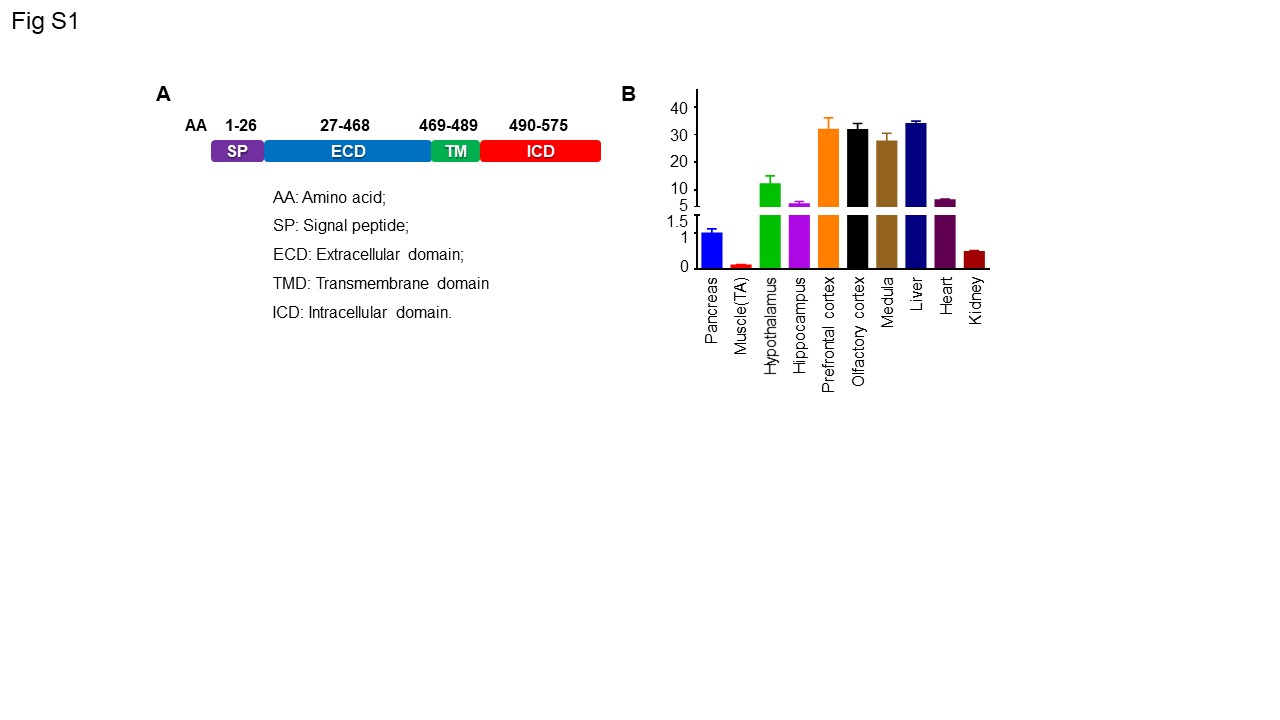

Supplement: Supplementary Figure 1 — Tmem108 expression profile in several indicated tissues in mice. A. Cartoon indicating TMEM108 domains in amino acid sequence. B. Relative expression Tmem108 in indicated tissues in 3-moth-old wild-type mice by Real-time PCR, Gapdh expression as internal control, Tmem108 relative expression in the pancreas was defined as one, wild-type mice, n=5. [file Image_1.jpeg]

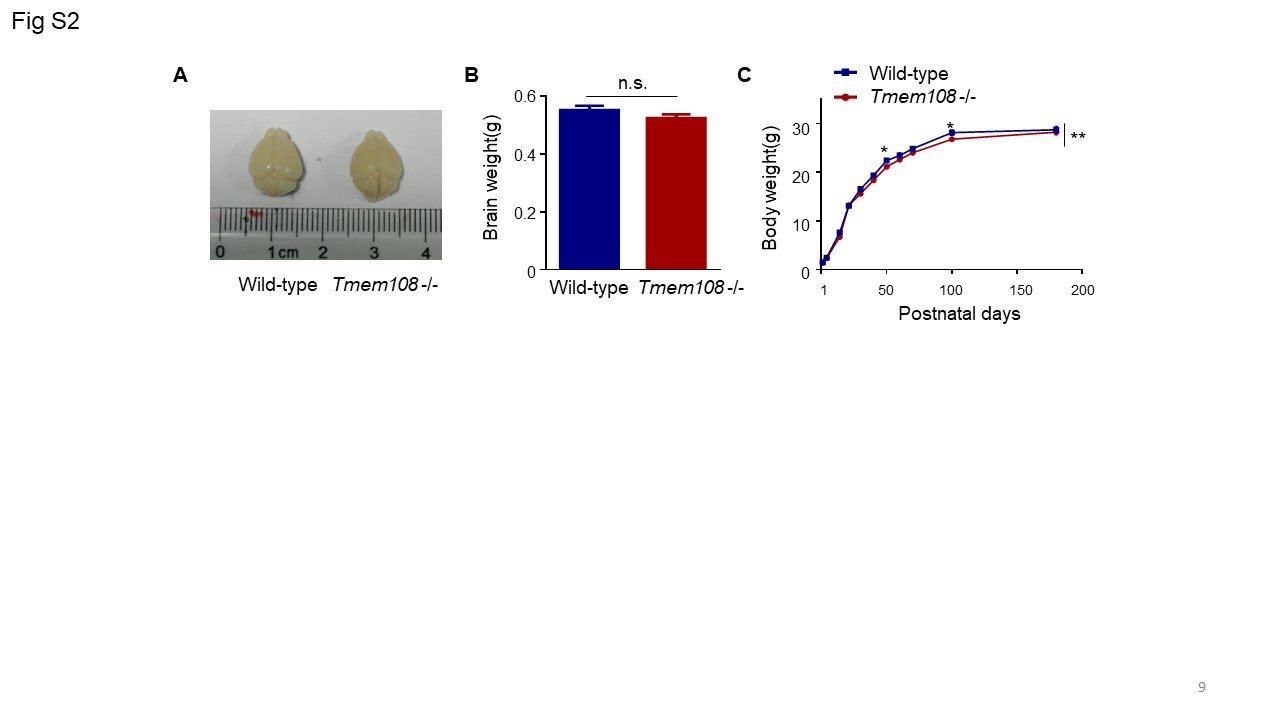

Supplement: Supplementary Figure 2 — Altered body growth curve of Tmem108 mutant mice. A. Representative brain figures of the adult mice (2 months). B. No alteration of brain weight in Tmem108 mutant (Tmem108 -/-) mice, wild-type mice as the control (Values are mean ± SEM, n = 12 2-month-old mice per group, unpaired t-test, n.s., not significant). C. Growth of Tmem108 mutant mice was slower than the control mice. (mice per group, n = 12, Sidak’s multiple comparisons test was used to compare the two groups accompanying with two-way ANOVA, *p < 0.05, **p < 0.01). [file Image_2.jpeg]
